# Unsolved mystery of Fas: mononuclear cells may have trouble dying in patients with Sjögren’s syndrome

**DOI:** 10.1186/s12865-023-00544-5

**Published:** 2023-06-23

**Authors:** Irena Lindrova, Martina Kolackova, Tereza Svadlakova, Radka Vankova, Marcela Chmelarova, Michaela Rosecka, Eva Jozifkova, Martin Sembera, Jan Krejsek, Radovan Slezak

**Affiliations:** 1 Department of Dentistry, Faculty of Medicine in Hradec Kralove, Charles University and University Hospital, Sokolska 581, 500 05 Hradec Kralove, Czech Republic; 2 Department of Clinical Immunology and Allergology, Faculty of Medicine in Hradec Kralove, Charles University, Simkova 870, 500 03 Hradec Kralove, Czech Republic; 3 Institute of Clinical Biochemistry and Diagnostics, University Hospital, Sokolska 581, 500 05 Hradec Kralove, Czech Republic; 4 Department of Biology, Jan Evangelista Purkyne University, Za Valcovnou 1000/8, 400 96, Usti and Labem, Czech Republic

**Keywords:** Autoimmune disorders, Fas, Fas Ligand, CD95, CD95L, Sjögren’s syndrome, B cells, T cells, Monocytes, NSAIDs

## Abstract

**Background:**

Patients with Sjögren’s syndrome, like other patients with autoimmune disorders, display dysregulation in the function of their immune system. Fas and Fas Ligand (FasL) are among the dysregulated proteins.

**Methods:**

We studied Fas and FasL on IL-2Rα^+^ cells and in serum of patients with Sjögren’s syndrome (*n* = 16) and healthy individuals (*n* = 16); both from same ethnic and geographical background. We used flow cytometry and enzyme-linked immunosorbent for this purpose. We also measured the expression of *Bcl-2* and *Bax* by reverse transcription quantitative real-time PCR (RT-qPCR) and percentage of apoptotic and dead cells using Annexin V and 7-AAD staining in lymphocytes.

**Results:**

FasL was increased in patients’ T and B cells while Fas was increased in patients’ monocytes, T and B cells. No signs of increased apoptosis were found. sFas and sFasL in patients’ serum were increased, although the increase in sFasL was not significant. We suspect an effect of non-steroidal anti-inflammatory therapy on B cells, explaining the decrease of the percentage Fas^+^ B cells found within our samples. In healthy individuals, there was a noticeable pattern in the expression of FasL which mutually correlated to populations of mononuclear cells; this correlation was absent in the patients with Sjögren’s syndrome.

**Conclusions:**

Mononuclear cells expressing IL-2Rα^+^ had upregulated Fas in Sjögren’s syndrome. However, the rate of apoptosis based on Annexin V staining and the *Bcl-2/Bax* expression was not observed in mononuclear cells. We suspect a functional role of abnormal levels of Fas and FasL which has not been cleared yet.

**Supplementary Information:**

The online version contains supplementary material available at 10.1186/s12865-023-00544-5.

## Background

Besides its physiological role (of inducing apoptosis), the Fas–Fas ligand (FasL) pathway was implicated in autoimmune diseases with diverse background, such as systemic lupus erythematosus (SLE) and multiple sclerosis. The outreach of this pathway tells us how complex autoimmune diseases are and that a patient’s body battles many changes when going from physiological state to the harming autoimmune responses [1–3]. Our study on Fas and FasL in cells expressing IL-2Rα is another small piece of information that explains involvement of these proteins in pathogenesis of Sjögren's syndrome, and that their function goes beyond the well-known role in apoptosis. Indeed, Fas-FasL interaction which leads to cell proliferation, autoantigen generation or reverse signalling pathway by FasL, has been described in detail [4–6]. Although apoptosis is one of the known mechanisms which can destroy exocrine glands, specifically acinar cells, results of the studies on lymphocyte apoptosis in Sjögren’s syndrome are conflicting [7, 8]. Mononuclear cells, primarily lymphocytes, infiltrate the exocrine glands and express FasL which in a common setting, induces apoptosis in cells carrying Fas. Both Fas and FasL were previously described to be abnormally expressed by various cells in patients with Sjögren’s syndrome [9, 10].

Many cells constitutively express Fas while the expression of FasL is usually restricted to the immune cells. In activated T cells, the upregulation of transcription factor IRF-1 as well as NF-κB is necessary for the expression of FasL [11, 12]. A binding site for NF-κB is present in the Fas gene too, thus resulting upregulation of both FasL and Fas in activated cells is imaginable [13].

Question remains whether activated T and B cells in the patients with Sjögren’s syndrome undergo apoptosis after executing their function. When comes to Fas and FasL, there are many check points in which apoptosis can be suspended and diverted. Beginning with soluble forms of Fas and FasL (sFas and sFasL) that may not only interfere with apoptosis but also support cell proliferation, neat interplay between downstream proteins determines the result of Fas signalling [14–17]. sFas and sFasL were found dysregulated in the patients with Sjögren’s syndrome; in some studies, their abnormal level was even correlating to clinical features and prognosis [18–20].

FasL-induced apoptotic pathway commonly involves death-inducing signalling complex (DISC) formation by recruiting the adaptor protein, Fas-associated protein with death domain, FADD, to death domains of the oligomerized receptor and the subsequent caspase 8/10 activation [21]. However, the ligation of Fas with sFasL that is generated by cleavage of membrane FasL by matrix metalloproteinase-7 (MMP7) does not lead to FADD and caspase-8 recruitment [22]. Instead, calcium-inducing domain of Fas interacts with phospholipase Cγ1 [3]. The molecular complex which is formed is referred to as motility-inducing signalling complex [23]. It has no apoptotic function, but it promotes (not only) Th17 endothelial transmigration. Therefore, high level of sFasL is likely to play the pathological role by enhancing Th17 migration to afflicted tissues in patients with autoimmune diseases. Disruption of this pathway may represent a good target for the therapy [24].cFLIP (cellular FLICE-like inhibitory protein) is another regulatory protein that can be recruited along with caspase-8 to the DISC. In T cells, cFLIP is capable of both inhibition of apoptosis and support of proliferation [25]. Following the activation of TLRs, cFLIP is also an essential protein in proliferation of B cells [26]. Other regulatory proteins include Bcl-2 and Bax. If upregulated, Bcl-2 can prevent Bax oligomerization [27, 28].Only oligomerised Bax anchors in the mitochondrial membrane and induces the formation of pores through which cytochrome c and Diablo (SMAC) escape. Thus, Bax works as the pro-apoptotic protein while Bcl-2 acts as the anti-apoptotic protein [29, 30]. As such, they were used in this study.

Downstream activated caspase-3 is an executioner caspase in apoptosis but also represents another regulatory point as it can participate in the generation of an excessive number of autoantigens, such as α-fodrin and cleaved poly signalling (ADP-ribose) polymerase, which both accompany autoimmune diseases, including Sjögren’s syndrome [5, 31].

As mentioned before, upregulation of (unmutated) Fas and FasL does not necessary lead to apoptosis and the Fas signalling pathway can not only result in the increased survival and proliferation of autoreactive immune cells but also autoantigen generation. Although several polymorphisms have been reported in association with autoimmune diseases, epigenetic modulation of gene expression and the interplay between pathways may take part in various outcomes of the signalling, and hence the severity of clinical symptoms of a given disease [32–34].

In Sjögren’s syndrome, unusual expression of IL-2/(s)IL-2Rα has been also described. IL-2Rα represents the low-affinity receptor with no signalling function while combining IL-2Rα with the β and γ chain generates high-affinity trimeric complex. Only cytoplasmic part of IL-2β transduces signal following IL-2 binding [35]. Among transcription factors activated by the IL-2 signalling pathway belongs STAT5 with differing level of phosphorylation observed in peripheral blood cells of Sjögren’s syndrome patients [36, 37].

IL-2Rα is expressed in lymphocytes as well as monocyte/macrophages population [38–41]. As for CD4^+^ and CD8^+^ T cells, IL-2Rα is only minimally expressed unless their TCR is stimulated [35]. In contrast, Tregs stably express trimeric IL-2R at high level and can respond to the low concentration of IL-2 thus outcompeting other lymphocytes that increase IL-2Rα only transiently during activation [42]. Both CD4^+^ and CD8^+^ T cells secrete high concentrations of IL-2 upon activation. IL-2 has an autocrine effect on these cells [43, 44]. On the other hand, Tregs are incapable of IL-2 production and depend on paracrine production [45]. Their response on IL-2 stimulation differs in Sjögren’s syndrome patients [37].

Some B cells also express IL-2Rα. These subsets carry considerably higher level of immunoglobulins on their surface and work better as antigen presenting cells than the subsets without IL-2Rα [46]. Therefore, B cells expressing IL-2Rα are suspected of playing a part in the pathology of Sjögren’s syndrome.

High serum levels of sIL-2Rα are found during lymphocyte activation and involvement of this protein has been reported in several autoimmune disease, including Sjögren’s syndrome [37, 47]. Fas/FasL and IL-2Rα/IL-2 correlate with severity of Sjögren’s syndrome and the efficacy of its treatment [37, 47, 48] Anti-inflammatory drugs (NSAIDs) can interfere with proliferation and apoptosis via several mechanisms [49]. These involve activation of caspases by intrinsic and extrinsic death receptor mediated pathway. Both induced pathways are likely to converge in mitochondria [50]. The fact that NSAIDs can increase apoptosis of lymphocytes with autoimmune features may be beneficial for patients with Sjögren’s syndrome.

In this article, we followed the expression of Fas, FasL, and IL-2Rα in mononuclear cells to either confirm or exclude their abnormal levels in patients with Sjögren's syndrome. High expression of Fas and FasL is connected to cell apoptosis. As we confirmed increased level of Fas and FasL in given patients' cells, we also tested the rate of apoptosis. As no increase rate of apoptosis was found among mononuclear cells, we specifically investigated the expression of Bcl-2 and Bax in B cells and CD4 T cells as described further.

## Methods

### Patients and controls

A group of 16 patients with primary Sjögren's syndrome participated in this study. All patients fulfilled the European-American consensus group criteria (AECC). The diagnosis of Sjögren's syndrome was driven on the basis of a routine evaluation of the patient’s symptoms and laboratory results (auto-antibody analysis, test of salivary flow rate, Schirmer’s test, medical records, etc.) performed at the Departments of Dentistry, Immunology and Allergy, Rheumatology and Ophthalmology at the University Hospital in Hradec Kralove, CZ, Table 1.

**Table 1:** Demographic and clinical data

	**Controls (16)**	**Patients (16)**		**Controls (16)**	**Patients (16)**
Men/Women	1/15	2/14	**Autoantibodies**	**0**	**15**
Age (years)	53.5	56	Anti-Ro	0	13
Leukocytes (× 10^9^/l)	6.8	4.85	Anti-La	0	11
Xerophthalmia	0	16	**Decreased salivary flow**	**N/A**	**8**
Xerostomia	0	15	**Schirmer’s test**	**N/A**	**16**
Dysphagia	0	10	**MSG biopsy**	**N/A**	**11**
TMJ disorders	0	8			
Thyroid dysfunction	0	4			
Arthritis	0	4			
Fatigue	0	11			
Weight loss	0	4			
Raynaud’s phenom	0	3			
NSAIDs	0	5			
Cyclosporin A	0	4			
Corticosteroids	0	5			
Antimalarials	0	2			

*Abbreviations*: *MSG biopsy* Minor salivary gland biopsy, *NSAIDs* Non-steroidal anti-Inflammatory drugs, *TMJ disorder* Temporomandibular joint disorder, *AUTOANTIBODIES* Anti-Ro, Anti-La, autoantibodies against ribonucleoproteins. Positive test of decreased salivary flow: saliva volume less than 8 ml/30 min (basal salivation (15 min)/stimulated salivation (15 min)). Positive Schirmer’s test: tear production less than 5 mm of the testing paper in 5 min. Positive minor salivary gland biopsy: focus score (FS) more or equal 1 (FS is defined as a count of lymphocytic foci containing more than 50 mononuclear cells per 4 mm^2^ tissue biopsy); these three tests were not applied in the control group

Age and number of leukocytes are displayed as median values. All other parameters are described by the number of positive cases. Xerophthalmia and xerostomia were characterised as subjective perception of eye and mouth dryness, respectively

The control group consisted of 16 healthy individuals who matched the patients in gender and age. The participants in the control group did not use any medications.

Both patients and controls agreed with their voluntary participation in this study and signed an informed consent.

Both groups were of the same ethnicity and recruited from the same geographical area. Number of subjects in the groups was assessed as described in Supplementary file.

### Samples

Blood samples were collected into Vacutainer tubes with heparin, tubes with EDTA, and tubes containing a thrombin additive (BD, UK). Anti-coagulated blood was used for flow cytometry and RT-qPCR immediately after the collection, while the serum was separated from blood elements by centrifugation at 1000 g for 15 min and frozen at -70 °C prior to analysis.

### Lymphocyte separation and RT-qPCR

We used negative separation (RosetteSep, StemCells Technologies, USA) with 1.081 g/cm^3^ density gradient medium (DM-L, StemCells Technologies, USA) to isolate the lymphocytes from EDTA-treated peripheral blood samples. In this population, the CD4^+^ T cells and B cells were further separated using CD4- and CD19-specific microbeads (PluriSelect, Germany). The expression levels of *Bax*, and *Bcl-2* mRNA were quantified in CD4^+^ T cells and B cells as described below. To standardise the results, the expression was compared to those of two housekeeping genes expression, *GAPDH* and *HPRT*. The total RNA was extracted from cells in TRIZOL using phenol–chloroform extraction. Approximately 1 μg of RNA was transcribed into cDNA using random primers and M-MLV Reverse Transcriptase, Rnase H Minus, Point Mutant (Promega, USA) in a total volume of 25 μl. Briefly, the samples and random primers were heated to 70 °C for 5 min and quickly chilled on ice. Five microlitres of 5xRT buffer, 5 μl of dNTPs (10 mM), 1 μl of RNAsin, and 1 μl of MLV H-RT were added to the samples. The mixtures were incubated for 10 min at room temperature and then heated to 47 °C for 50 min and 75 °C for 15 min in a standard thermal cycler (GeneAmp 9700, Applied Biosystems). TaqMan Gene Expression Master Mix and TaqMan Gene Expression Assays were used for amplification (Life Technologies, USA). Each reaction consisted of 45 cycles in a Rotor-Gene 6000 instrument (Corbett Live Science). The parameters for PCR were as follows: 50 °C for 2 min, 90 °C for 10 min and 45 cycles at 95 °C for 15 s and at 60 °C for 60 s.

### Mononuclear cell separation, flow cytometry, and ELISA

Mononuclear cells were collected from blood samples treated with EDTA by density gradient centrifugation. We used 1.077 g/cm^3^ medium (PBS-diluted 1.124 g/cm^3^ Easycoll, Biochrom, Germany) for the separation. The platelets were further removed on 1.063 g/cm^3^ Easycoll at 400 g for 15 min. The cells were washed with pre-binding buffer (140 mM NaCl, 4 mM KCl, 0.75 mM MgCl_2_, 10 mM HEPES; pH 7.2 – 7.4) and re-suspended in binding buffer (pre-binding buffer recipe plus 2.5 mM CaCl_2_). The cells were stained with Annexin V FITC (NeXins Research, Netherlands) and 7-AAD (BD, USA) for 15 min at room temperature in the dark. We measured the samples on a FACSCalibur flow cytometer (BD, USA).

The expression of Fas and FasL in various cell populations was measured in whole blood samples treated with heparin. The peripheral blood samples were stained with the following combination of antibodies: anti-Fas FITC/IL-2Rα PE/CD19 PerCP/CD4 APC and anti- IL-2Rα FITC/Fas Ligand PE/CD19 PerCP/CD4 APC. Fluorescence minus one (FMO) control determined the positive expression of the aforementioned markers, but we also used isotype controls to exclude non-specific binding. Cells expressing markers at a low density were also considered positive. All antibodies were purchased from Exbio, CZ. Following staining, the red blood cells were lysed using an isotonic solution of NH_4_Cl, centrifuged, and immediately measured on the flow cytometer. The adequate performance of the flow cytometer was regularly verified using CaliBrite beads and FACSComp (BD, USA). CellQuest (BD) was used to acquire data. The collected data were batch-analysed with the FlowJo 8.7 software (TreeStar, USA). The expression was characterised with the median fluorescence intensity, MFI. We used flow cytometry as a semi-quantitative method. The result was measured using the same instrument setting, which enabled us to compare patients and controls. Therefore, all charts display relative values and do not allow inter-laboratory comparisons.

An enzyme-linked immunosorbent assay (ELISA) was performed using the sFas ELISA kit and the sFasL ELISA kit (R&D Systems, USA). The detection range of the assay specific for sFas was 15.6 – 2000 pg/ml. The manufacturer declared no cross-reactivity between anti-sFas antibodies and other members of the TNFR family. The detection range of the assay specific for sFasL was 7.8 – 1000 pg/ml. The manufacturer observed no cross-reactivity between anti-sFasL antibodies and members of the TNF family other than FasL.

### Statistical analyses

The normality of the sampled data was tested using the Shapiro–Wilk test. The homogeneity of variances was determined with Levene’s test. Normally distributed and homoscedastic data were compared using a t-test; otherwise, a Mann–Whitney U Test or Kolmogorov–Smirnov Test was used. The relationship between variables was assessed using the Pearson correlation coefficient. Categorical variables were evaluated with Fisher’s exact test. The data were tested at a significance level of 5%. We used Statistica 10 (StatSoft, USA) to perform the tests. SPSS 21 (IBM, USA), procedure GENLINMIXED was used for generalized linear mixed models. Statistica 10, GraphPad Prism 8 (GraphPad Software, USA) and Microsoft Excel (version 14, 2010 Microsoft Corporation, USA) were used to plot graphs. Graphs that show individual cases only display median values. All other graphs display median values, quartiles, and non-outliers, unless stated otherwise. Asterisks mark the level of the statistical significance. * is for *P* ≤ 0.05, ** for P ≤ 0.005, and *** marks *P* ≤ 0.001.

## Results

### Study participants

Clinical features are summarized in Table 1. Regarding other clinical symptoms, thyreopathy was noted in 4 patients (25%), joint paint in 11 patients (68.8%), temporomandibular disorders in 8 patients (50%), recurrent parotitis in 7 patients (43.8%), and chronic fatigue in 11 patients (68.8%).

We adapted the point scoring system for the clinical evaluation of Sjögren’s syndrome as described.

1. Serology.

- Positive anti-SSA/Ro and/or anti SSB/La = score of 5.
- Negative anti-SSA/Ro and anti-SSB/La = score of 0.

2) Minor salivary glands biopsy.

- FS ≥ 1 as defined in Table 1 = score of 4.
- Normal salivary gland biopsy = score of 0.

3) Oral examination*

- Decreased salivary flow 1 ≤ 8 ml as defined in Table = score of 1.
- Normal salivary flow = score of 0.

4) Ocular examination.

- CFS ≥ 3 or Schirmer’s test ≤ 5 mm/5 min or BUT ≤ 5 s = score of 1.
- Normal or CFS < 3 and/or Schirmer’s test > 5 mm/5 min and/or BUT > 5 s = score of 0.

*SPECT imaging of salivary gland was not performed. Abbreviations: BUT: breakup time, CFS: cornea fluorescein staining, FS: focus score.

Similar comparison as described for patients and controls was used for subgroup of patients with low and high score (Table 2). We compared cell-associated characteristics (expression of Fas and FasL) as well as serum proteins (sFas and sFasL) using t-tests, but no differences were found.

**Table 2:** Scoring

	Patients (no.)
Low score (5–7)	8
High score (10–11)	8

### Lymphocytes and monocytes

**Fig. 1:**
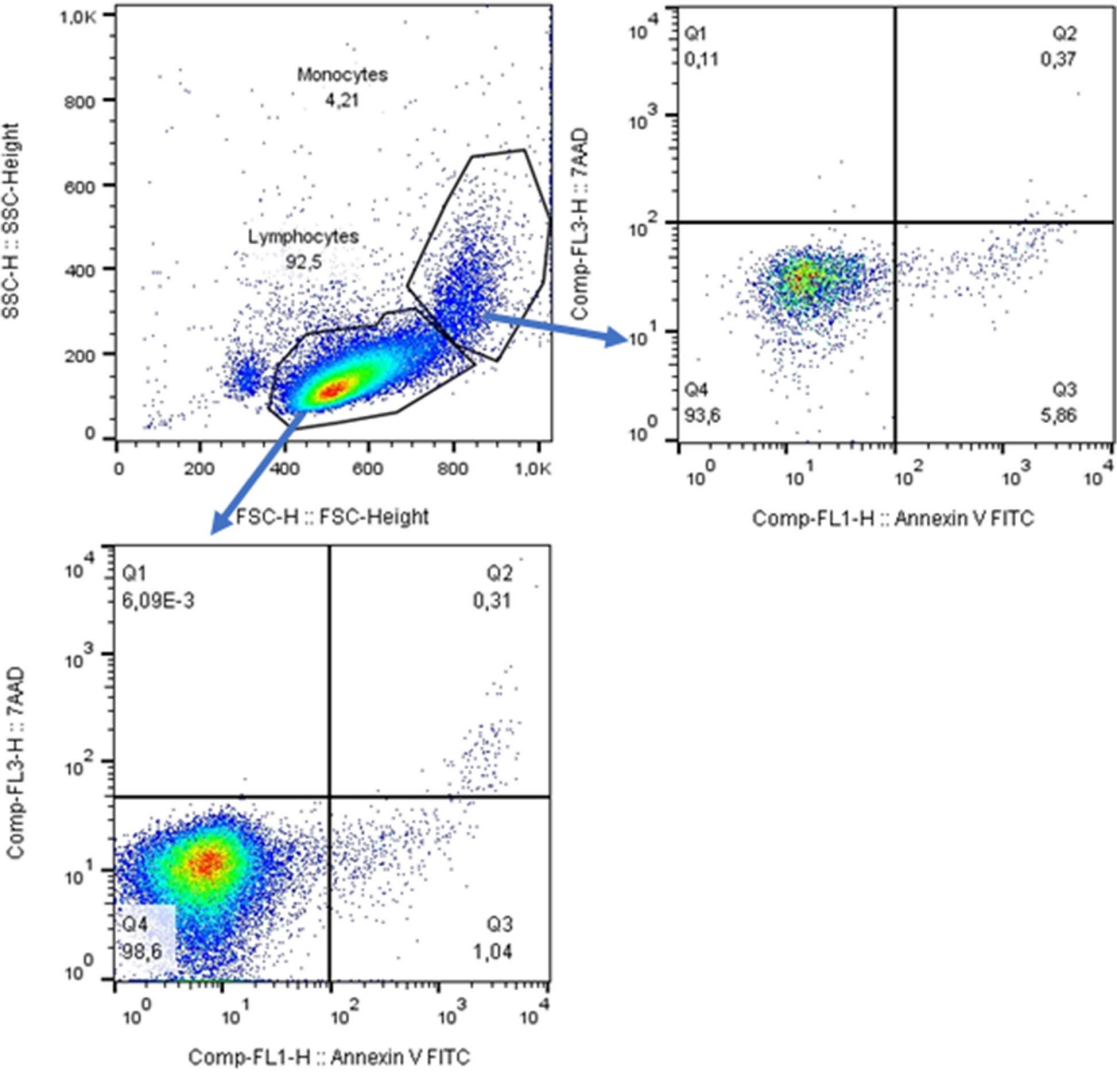
Flow cytometry analysis of early apoptotic and late apoptotic or dead cells

**Fig. 2:**
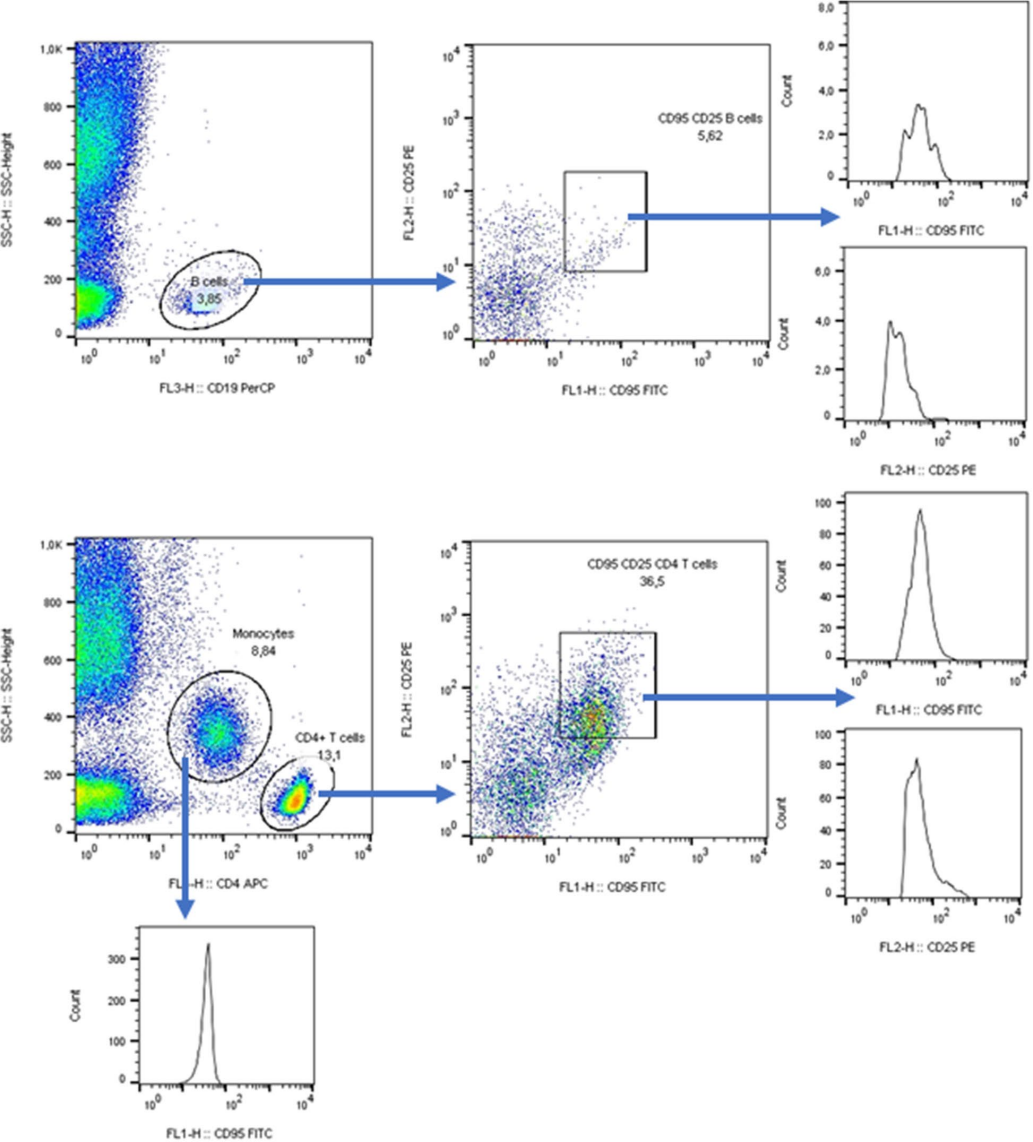
Gating strategy for the analysis of Fas in IL-2Rα^+^ B cell, IL-2Rα^+^ CD4^+^ T cells, and monocytes in the peripheral blood samples of patients and controls. Expression of IL-2Rα was also evaluated

**Fig. 3:**
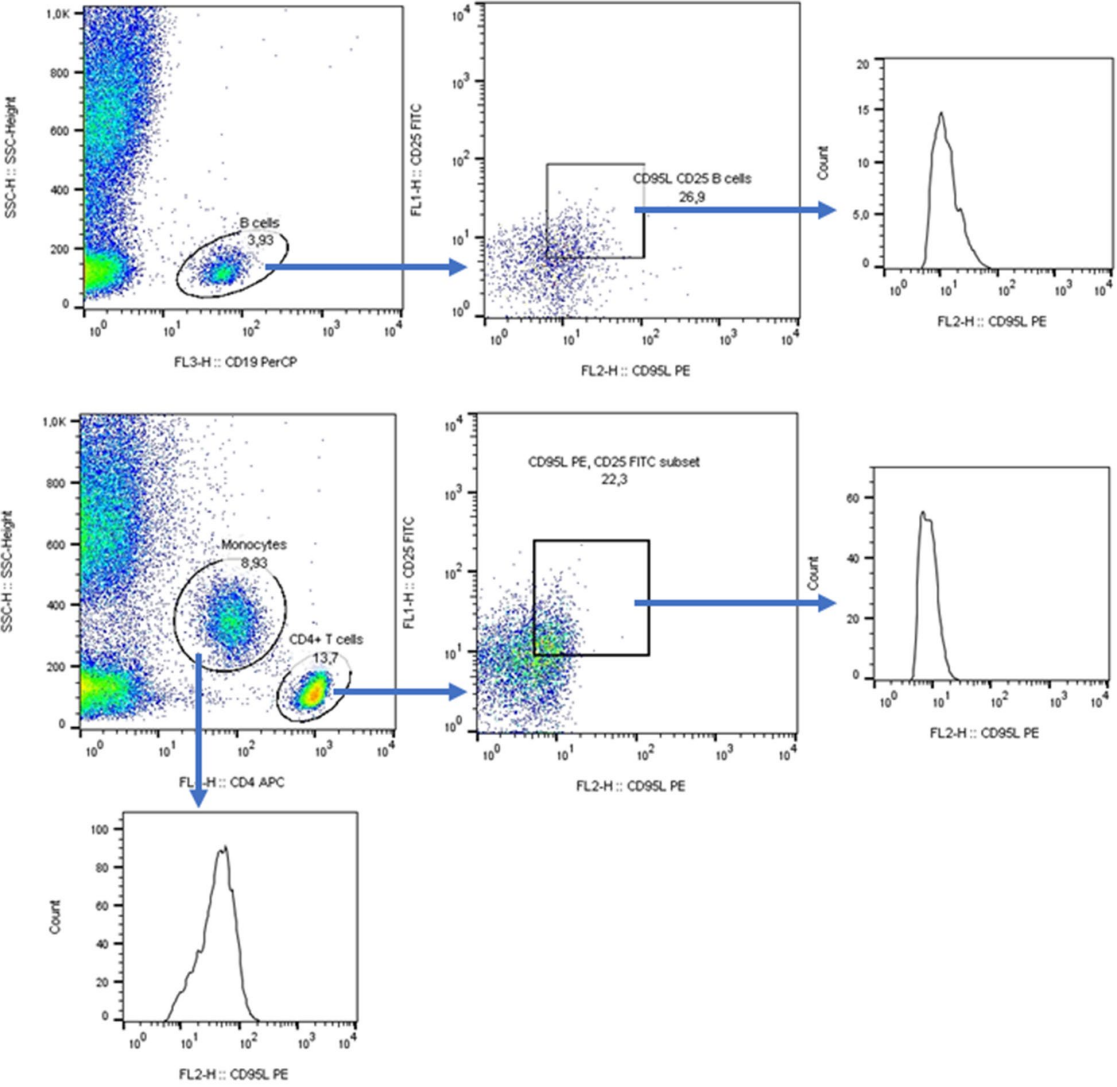
Gating strategy for the analysis of FasL in cell populations as in Fig. 2

Populations of leukocytes were distinguished on basis of forward-scattered and side-scattered light characteristics. These parameters were sufficient to separate lymphocytes and monocytes in samples of isolated mononuclear cells, Fig. 1. In the whole blood samples, cell surface marker CD4 was used to distinguish T cells and monocytes. CD 19 separated B cells from other lymphocytes (Fig. 2 and 3).

### Apoptosis

Annexin V and 7-AAD staining revealed a low frequency of apoptotic and dead cells in both patients and controls. The percentage of apoptotic lymphocytes (1.17% in controls, 0.87% in patients) and percentage of apoptotic monocytes (4.35% in controls, 4.57% in patients) did not significantly differ between groups. The results were similar for the percentage of late apoptotic and dead lymphocytes (0.27% in controls, 0.24% in patients) and monocytes (0.38% in controls, 0.37% in patients), Fig. 1.

Expression of *Bcl-2* and *Bax* (analysed by RT-qPCR), as well as the ratio of *Bax* to *Bcl-2* did not differ between patients and controls, Table 3.

**Table 3:** Ratio of *Bax* to *Bcl-2* (normalised to *HPRT*)

	**Controls**		**Patients**	
	Median	(Min – Max)	Median	(Min – Max)
**CD4**^**+**^** T cells**	0.62	(0.53 – 0.95)	0.6	(0.55 – 1.06)
**B cells**	0.69	(0.62 – 0.78)	0.69	(0.67 – 0.84)

Number of repeats: 3

### Expression of Fas and FasL

**Fig. 4:**
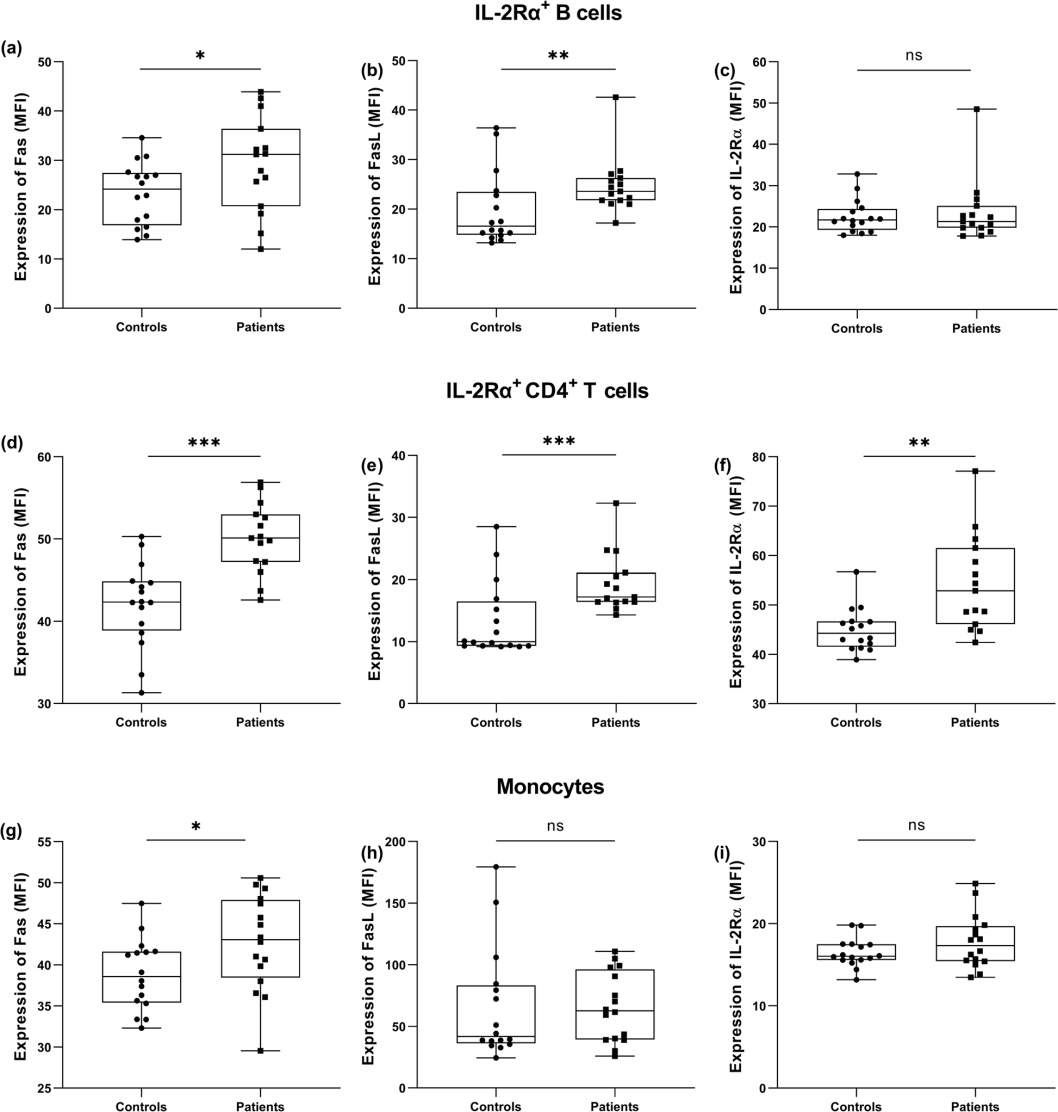
The comparison of Fas and FasL expression between controls and patients with Sjögren’s syndrome. **a** Expression of Fas in IL-2Rα^+^ B cells, t-test. **b** Expression of FasL in IL-2Rα^+^ B cells, Mann–Whitney U Test. **c** Expression of IL-2Rα in IL-2Rα^+^ B cells, Mann–Whitney U Test. **d** Expression of Fas in IL-2Rα^+^ CD4^+^ T cells, t-test. **e** Expression of FasL in IL-2Rα^+^ CD4^+^ T cells, Mann–Whitney U Test. **f** Expression of IL-2Rα in IL-2Rα^+^ CD4^+^ T cells, Kolmogorov–Smirnov Test. **g** Expression of Fas in monocytes, t-test. **h** Expression of FasL in monocytes, Mann–Whitney U Test. **i** Expression of IL-2Rα in monocytes, t-test

The expression of Fas and FasL (expressed as MFI) was analysed in association with IL-2Rα (Fig. 2, 3). In Sjögren’s syndrome patients, the expression of Fas was increased in all observed populations of cells, whereas FasL was only increased in lymphocytes (explained in further details). CD4^+^ T cells were the sole population with increased expression of IL-2Rα (Fig. 4).

The expression of Fas in IL-2Rα^+^ B cell and IL-2Rα^+^ CD4^+^ T cells was higher in patients than in controls (*P* = 0.037 and *P* < 0.001, respectively), Fig. 4a, d. The expression of Fas was also significantly higher in patients’ monocytes (*P* = 0.04), Fig. 4g.

The expression of FasL in IL-2Rα^+^ B cells and IL-2Rα^+^ CD4^+^ T cells was higher in patients than controls (*P* = 0.008 and *P* = 0.001, respectively), Fig. 4b, e. However, the expression of FasL in monocytes was not significantly different when comparing patients and controls (*P* = 0.44), Fig. 4h.

The expression of IL-2Rα was not different for B cells and monocytes (*P* = 0.867 and *P* = 0.151, respectively), Fig. 4c, i, but in CD4^+^ T cells, IL-2Rα was higher in patients (*P* < 0.025), Fig. 4f.

**Fig. 5:**
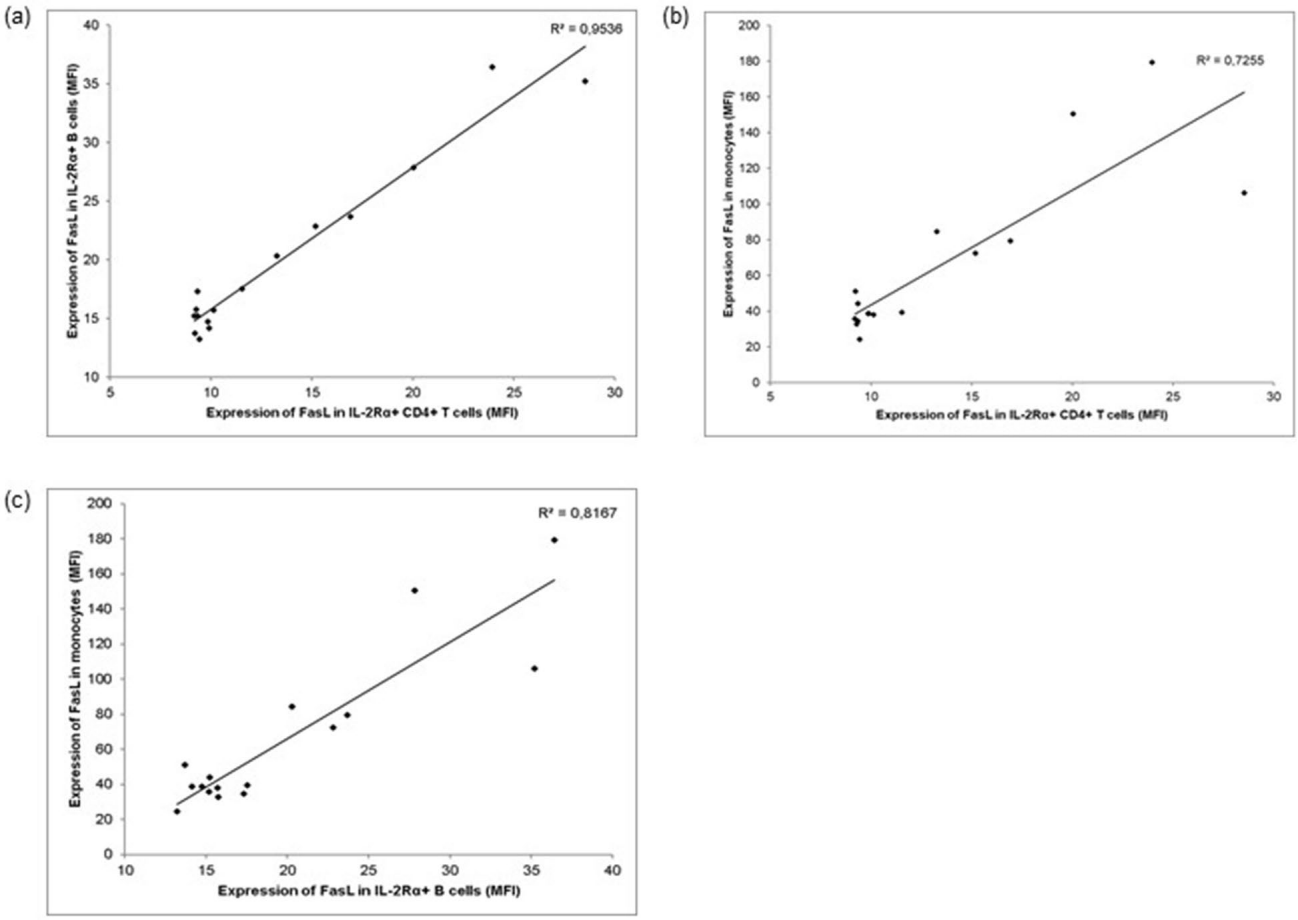
Correlation of the expression of FasL in the control group. **a** Correlation between IL-2Rα^+^ B cells and FasL in IL-2Rα^+^ CD4^+^ T cells. **b** Correlation between monocytes and FasL in IL-2Rα^+^ CD4^+^ T cells. **c** Correlation between IL-2Rα^+^ B cells and monocytes

We found a relationship between the expression of FasL on the cell membrane of lymphocytes and monocytes of controls but not in the patients. The expression of FasL correlated between the following three cell populations: FasL^+^ IL-2Rα^+^ B cells and FasL^+^ IL-2Rα^+^ CD4^+^ T cells (R^2^ = 0.95, *P* < 0.001), FasL^+^ IL-2Rα^+^ CD4^+^ T cells and monocytes (R^2^ = 0.73, *P* < 0.001), monocytes and FasL^+^ IL-2Rα^+^ B cells (R^2^ = 0.82, *P* < 0.001), Fig. 5a, b, c.

### Percentage of Fas^+^ and FasL^+^ cells

The percentage of monocytes, IL-2Rα^+^ B cells, of IL-2Rα^+^ CD4^+^ T cells did not differ in controls and patients (*P* = 0.133, *P* = 0.769, and *P* < 0.1), data not shown. However, there were differences in percentage of cells expressing Fas and FasL when comparing patients and controls, Fig. 2, 3.

**Fig. 6:**
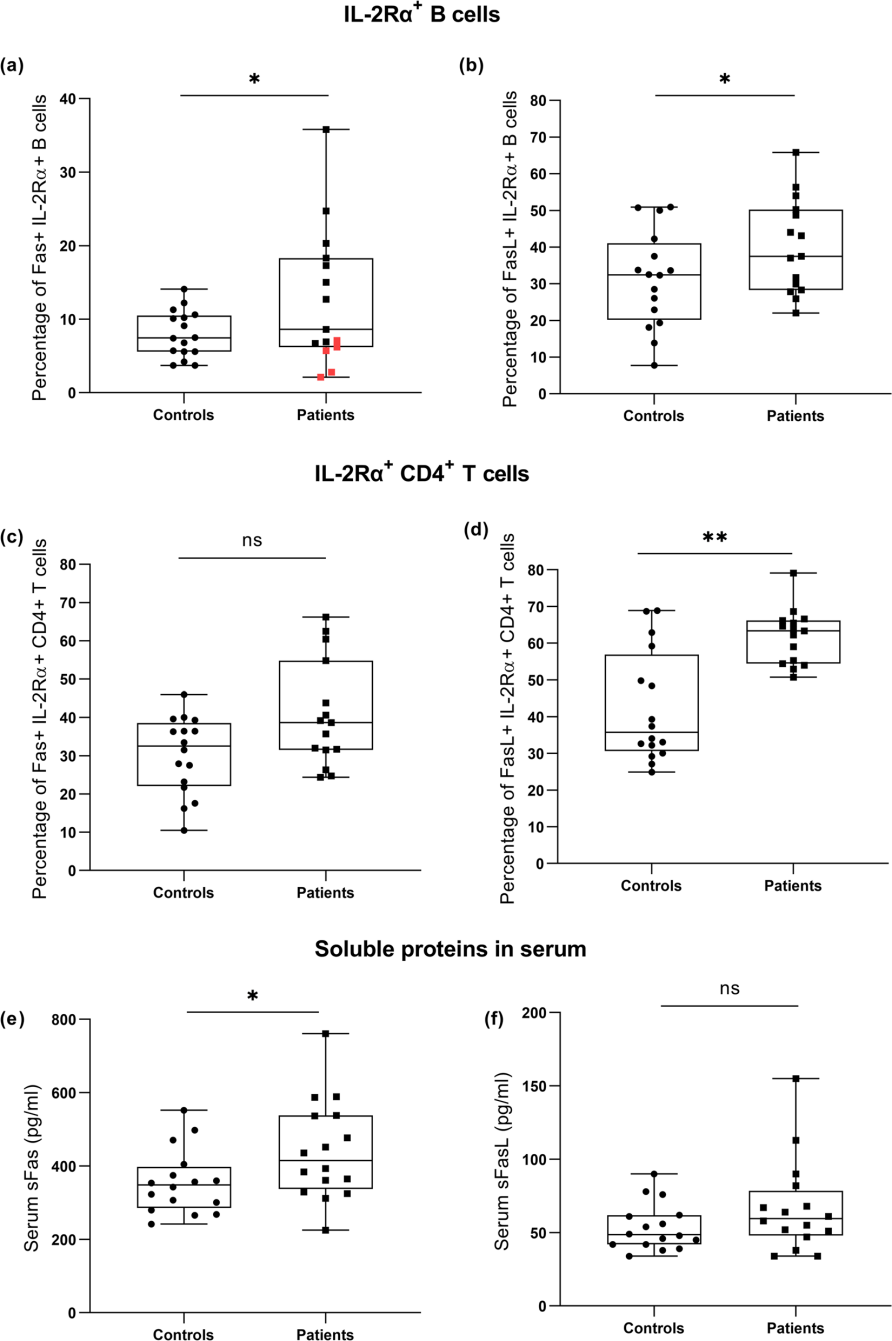
The comparison of Fas and FasL- positive lymphocytes and serum levels between controls and patients with Sjögren’s syndrome. **a** Percentage of Fas^+^ IL-2Rα^+^ B cells, patients using NSAIDs are marked by red squares, Kolmogorov–Smirnov Test. **b** Percentage of FasL^+^ IL-2Rα^+^ B cells, t-test. **c** Percentage of Fas^+^ IL-2Rα^+^ CD4^+^ T cells, t-test. **d** Percentage of FasL^+^ IL-2Rα^+^ CD4^+^ T cells, Kolmogorov–Smirnov Test. **e** Serum level of Fas, t-test. **f** Serum level of FasL t-test

The percentage of Fas^+^ IL-2Rα^+^ B cells was significantly increased in patients (*P* < 0.05), Fig. 6a. Strikingly, all five patients that used NSAIDs had lower percentage of Fas^+^ IL-2Rα^+^ B cells than the other eleven patients who did not use NSAIDs (median value of 18.8% vs. 49.6%, *P* < 0.001). As a result, the percentage of Fas^+^ IL-2Rα^+^ B cells in patients using NSAIDs was similar to the percentage of these cells in controls (median value of 23.5%), Fig. 6a.

**Fig. 7:**
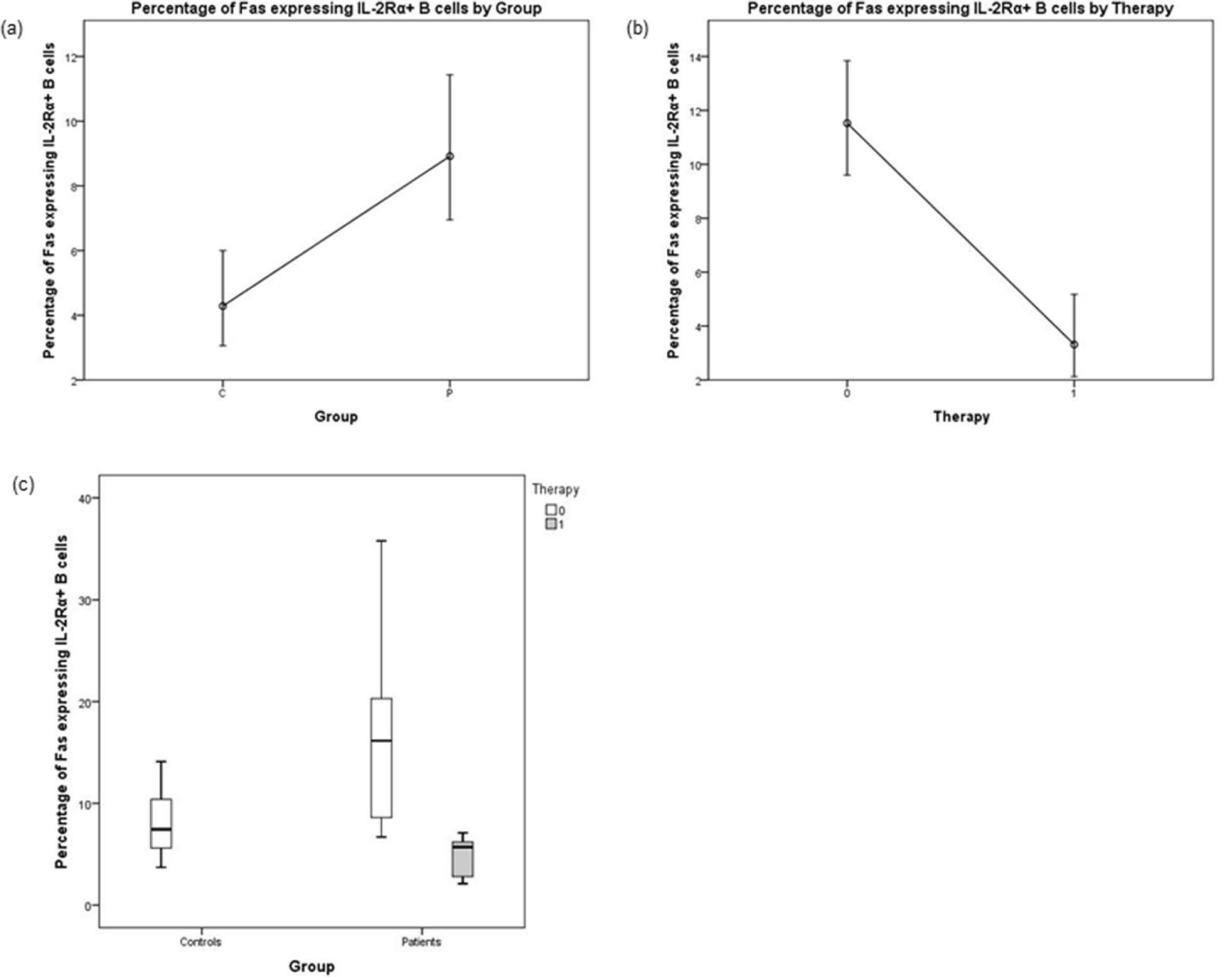
Generalized linear mix models. **a** Estimated marginal means for groups. **b** Estimated marginal means for therapy. **c** The percentage of Fas expressing IL-2Rα + B cells in patients (P) with therapy (1), without therapy (0), and controls (C)

To exclude the effect of NSAIDs, we used generalized linear mix models. In this model, the percentage of Fas expressing IL-2Rα^+^ B cells was the target, thus the patients and control group were the fixed effects, and the therapy was the random effect. Patients differed from controls (F = 16.826; df1 = 1; df2 = 28; *P* < 0.001). Figure 7a. When considering NSAIDs as the fixed effect in order to compare patients with and without the therapy, both groups differed significantly (F = 26.344; df1 = 1; df2 = 28; *P* < 0.001), Fig. 7 b, c.

The percentage of Fas^+^ IL-2Rα^+^ CD4^+^ T cells was increased in patients but not significantly (*P* = 0.096), Fig. 6c.

The percentage of FasL^+^ IL-2Rα^+^ B cells and FasL^+^ IL-2Rα^+^ CD4^+^ T cells was higher in patients than controls (*P* = 0.011 and *P* < 0.005, respectively), Fig. 6b, d.

### Soluble Fas and FasL

Analysis of the sera by ELISA revealed the significantly higher concentration of sFas in patients than in controls (*P* = 0.043), Fig. 6e. Furthermore, the concentration of sFasL was higher in patients than in controls (median value of 60 pg/ml and 49 pg/ml, respectively), but this difference was not significant (*P* = 0.152), Fig. 6f.

## Discussion

Most of autoimmune diseases cannot be linked to a single gene mutation. This is depicted by the animal model of MRL-Fas ^lpr^ mice where mice developed Sjögren’s-like and systemic lupus erythematosus-like symptoms due to mutational changes of the *Fas* gene. However, no such profound correlations between *Fas* polymorphisms or mutations and Sjögren's syndrome or systemic lupus erythematosus in humans has been found. It is more likely that epigenetic modifications and interactions between signalling pathways impact Fas and FasL.

Beside our study on Fas and FasL expression, there are other studies which report dysregulated production of Fas, FasL and their soluble forms in Sjögren’s syndrome [20, 48]. However, the results are conflicting. While the study by Benchabane et al., 2022 reported upregulation of sFasL [48], Luo et al. 2017 found completely opposite trend [20], and Nozawa’s group found no trend [19]. Similarly, Sahin et al., 2007 and Ogawa et al.,1999 described no change in sFas, but studies carried by Benchabane et al., 2022, Nozawa et al. 1997 and us (Fig. 6e) found the increase of sFas in the patients [19, 48, 51, 52]. Some putative discrepancies, such as the expression of Bcl-2 in Sjögren’s syndrome, are the result of different methodical approaches (e.g., serum and salivary glands versus peripheral blood cells).

Several studies have investigated apoptosis in Sjögren’s syndrome [53, 54]. Ohlsson and co-workers considered apoptosis a rare event in these patients [8]. They measured a low rate of apoptosis in populations of CD4^+^ and CD8^+^ T cells as well as in mononuclear cells that infiltrated salivary glands of the patients. Ohlsson’s finding was complemented by the research of Busamia and co-workers, who found a high degree of proliferation among infiltrating lymphocytes in salivary glands located in the lower lip of patients with Sjögren’s syndrome [55]. These data seem to conflict other studies that found an increased rate of apoptosis among the infiltrating T cells [52, 56]. Ohlsson’s observation corresponds with the results we found in peripheral blood lymphocytes, which also highly expressed Fas, but the rate of apoptosis was the same as that in control samples.

Furthermore, our findings along with the Ohlsson’s suggest disruption of activation-induced cell death (AICD) [57]. AICD requires an increase of FasL and Fas in cells at the beginning of their activation, while the cells remain resistant to Fas-induced apoptosis. Nevertheless, these cells finally undergo apoptosis, thereby preventing an exaggerated immune response in healthy individuals [58]. This regulative mechanism seems not to be functional in Sjögren’s syndrome.

Our further observations also indicated a failure of Fas/FasL regulation. The first observation involved no correlation in FasL expression which was present in healthy individuals (Fig. 5). The other finding was related to the increased concentration of serum sFas in our patients (*P* = 0.04). In healthy individuals, the activation of Fas promotes the expression of full-length Fas via the activation of kinase FAST K, which indirectly controls the inclusion of exon 6 in the pre-mRNA [59, 60]. Only Fas isoforms that include exon 6 are expressed on the cell membrane. This mechanism, which leads to the expression of membrane Fas, can be diverted by repressors of the inclusion of exon 6. As a result, a soluble isoform of Fas is generated. Therefore, the expression of membrane Fas should negatively correlate with the expression of sFas. Although the activation of Fas may be responsible for the increase in Fas expression on the cell membranes, it does not explain the increase of sFas that was also found in the sera of our patients. The mechanism by which the increased levels of both isoforms of Fas are maintained and why the cells that abundantly express Fas are not removed in Sjögren’s syndrome remains to be identified.

Although the sample size of our study group was small, variability within this group was low, particularly in terms of ethnicity (all patients were Caucasians who were born and raised in the Czech Republic). Therefore, we can consider our observations valid for the specified population which unfortunately excludes Vietnamese and Roma people who represent minorities in CZ. However, regarding the therapy that our patients received, there are limits in interpretation and extrapolation of our results because different immunosuppressive medication further fractions the sample group. Nozawa and co-workers described a connection between steroid therapy and the decrease in serum sFas and sFasL in patients with systemic lupus erythematosus [19]. We observed a similar connection between NSAIDs and the decrease in the percentage of Fas^+^ IL-2Rα^+^ B cells (Fig. 6a), but no effect of other immunosuppressive therapy (cyclosporin A and corticosteroids) on Fas and FasL expression.

We can conclude that at the molecular level, there is an aberrant expression of various proteins, including Fas and FasL, and unusual activation of signalling pathways which cannot be solely attributed Sjögren’s syndrome [61]. Although scientists as well as clinicians are aware of familial clustering and co-occurrence of clinically different autoimmune diseases, especially in the case of connective tissue disorders, there is no direct link between genetic factors and Sjögren’s syndrome, [62, 63]. We can expect that patients with Sjögren’s syndrome who belong to distinct ethnic groups will suffer from the disease to various extent not only because of different genetic background but also because of different environmental triggers associated with the disease. These two factors are likely to bring discordances into observations of mechanisms which underly Sjögren’s syndrome and make difficult to pinpoint markers that reflect the disease and its progression [64, 65].

## Conclusions

In Sjögren’s syndrome, the rate of apoptosis of peripheral blood lymphocytes and monocytes does not seem changed, even though these cells highly co-express Fas and IL-2Rα. However, the lymphocytes also upregulate FasL, and thus, may induce apoptosis of other cells. Still, due to the conflicting results between various studies, Fas and FasL do not seem to be suitable markers of Sjögren’s syndrome and its progression.

## Supplementary Information

**Additional file 1.**

## Data Availability

The datasets used and analysed during the current study are available from the corresponding author on reasonable request.
