# Supplementary material for: Unsolved mystery of Fas: mononuclear cells may have trouble dying in patients with Sjögren’s syndrome
Source: BMC Immunol. 2023 Jun 23;24:12. doi: 10.1186/s12865-023-00544-5 (PMC10288785; doi:10.1186/s12865-023-00544-5)
Supplement: Supplementary file 1 — Additional file 1. [file 12865_2023_544_MOESM1_ESM.docx]

Supplementary file

Sample size (n_d_) was estimated on the basis of preliminary data of seven patients with Sjögren’s syndrome and seven controls, coming from flow cytometry measurements. d-score was calculated from the absolute value of difference between the means of the control and patient group $(\overline{X}$_I_ and $\overline{X}$_j_) divided by the pooled standard deviation (s_p_):

$$d=\frac{\left| \overline{X}_{i}-\overline{X}_{j} \right|}{s_{p}}$$

Sample size reflects total number of patients with primary Sjögren’s syndrome who regularly visited the Department of Dentistry (N=60) and might have consented to their participation in the study. Margin of error (M) is set on 5 % and s_r_ denotes relative standard deviation of the sample:

$$n_{d}=\frac{\frac{ⅆ^{2}\left( s_{r}-{s_{r}}^{2} \right)}{M^{2}}}{1+\left( \frac{ⅆ^{2}\left( s_{r}-{s_{r}}^{2} \right)}{M^{2}N} \right)}$$

Calculated sample size reached a range of values from 5 to 24 with the mean of 14 and it was not recalculated in the course of the study. The demandingness of methods such as concurrent Th and B cell isolation as well as sequential centrifugation (in order to remove platelets from annexin V staining) further restrained number of subjects involved.
